# GENLIB: new function to simulate haplotype transmission in large complex genealogies

**DOI:** 10.1093/bioinformatics/btad136

**Published:** 2023-03-17

**Authors:** Mohan Rakesh, Hélène Vézina, Catherine Laprise, Ellen E Freeman, Kelly M Burkett, Marie-Hélène Roy-Gagnon

**Affiliations:** School of Epidemiology and Public Health, University of Ottawa, Ottawa, Ontario, Canada; Projet BALSAC, Université du Québec à Chicoutimi, Chicoutimi, Québec, Canada; Département des sciences humaines et sociales, Université du Québec à Chicoutimi, Chicoutimi, Québec, Canada; Centre intersectoriel en santé durable, Université du Québec à Chicoutimi, Chicoutimi, Québec, Canada; Centre intersectoriel en santé durable, Université du Québec à Chicoutimi, Chicoutimi, Québec, Canada; Département des sciences fondamentales, Université du Québec à Chicoutimi, Chicoutimi, Québec, Canada; School of Epidemiology and Public Health, University of Ottawa, Ottawa, Ontario, Canada; Centre de Recherche, Hôpital Maisonneuve-Rosemont, Montréal, Québec, Canada; Department of Mathematics and Statistics, University of Ottawa, Ottawa, Ontario, Canada; School of Epidemiology and Public Health, University of Ottawa, Ottawa, Ontario, Canada

## Abstract

**Summary:**

Founder populations with deep genealogical data are well suited for investigating genetic variants contributing to diseases. Here, we present a major update of the genealogical analysis R package GENLIB, centered around a new function which can simulate the transmission of haplotypes from founders to probands along very large and complex user-specified genealogies.

**Availability and implementation:**

The latest update of the GENLIB package (v1.1.9) contains the new gen.simuHaplo() function and is available on the CRAN repository and from https://github.com/R-GENLIB/GENLIB. Examples can be accessed at https://github.com/R-GENLIB/simuhaplo_functions.

## 1 Introduction

Founder populations have been utilized extensively in the study of Mendelian diseases because they can have higher incidence rates of rare autosomal-recessive genetic diseases due to drift effects, e.g.: Gaucher disease, Tay–Sachs disease, and cystic fibrosis in the Ashkenazi Jewish population (Charrow 2004), or one of the over 30 identified autosomal-recessive diseases with elevated frequency in the Finnish population (Pastinen et al. 2001, Norio 2003). In founder populations, affected individuals are more likely to have the causal mutation on longer haplotypes that are homozygous by recent decent, aiding in mutation discovery (Bourgain and Genin 2005, Libiger and Schork 2007). Some founder populations have extensive records allowing for reconstruction of deep and large genealogies (Vézina and Bournival 2020, Ober et al. 2001, Falchi et al. 2004, Liu et al. 2007). Gene-dropping simulations (Maccluer et al. 1986, Chen et al. 2015) can be performed within these genealogies, wherein ancestral genotypes are passed down a fixed pedigree structure. For example, allele-dropping was used to study mutation frequencies in the Hutterite (Chong et al. 2012), and French-Canadian (Heyer 1999) founder populations.

Gene-dropping is not limited to dropping specific alleles. Transmission of genomic regions, chromosomes, or even the entire genome can be simulated. This type of simulation can provide important information on the distribution of genomic sharing and the probability of sharing a specific genomic segment among close or distant relatives, and can identify specific founders and transmission paths responsible for the observed sharing. However, in very large genealogies, these gene-dropping simulations are computationally feasible only if one does not consider the allelic state of any specific locus, but rather only the positions of recombination events and the origin (founder) of the segments bounded by the crossovers (Cheng et al. 2015). We have implemented such a gene-dropping simulation tool in the GENLIB genealogical analysis R (http://www.R-project.org/) package (Gauvin et al. 2015, R Core Team 2021). This new tool (named gen.simuHaplo) is fast even for large genomic regions and deep genealogies with many individuals because it does not consider any alleles, mutations, or phenotypes. To our knowledge, it is the first user-friendly simulation tool that can perform gene-dropping simulations of long genomic segments in very large and complex genealogies while allowing the ability to retrace all transmission paths.

## 2 Implementation and usage

### 2.1 Overview of GENLIB and new function implementation

GENLIB is an R package designed to analyze large genealogical datasets. The basic argument for all GENLIB functions is a “genealogy” object created by the gen.genealogy function from a matrix or data frame where each line describes an individual with the following information: identification number (ID), father ID, mother ID, and sex (see Supplementary Appendix S1 for more details). GENLIB functions can be grouped into four categories: (i) genealogical data management, (ii) data description and visualization, (iii) computation of relevant statistics (e.g. kinship coefficients for pairs of individuals), and (iv) simulations. More details on the GENLIB original functions can be found in Gauvin et al. (2015).

The new gen.simuHaplo function simulates genomic segments (hereafter interchangeably referred to as haplotypes) of user-specified lengths in specified or all probands of a genealogy. Meiosis in the parents of each individual is simulated using one of three possible models (see Supplementary Appendix S1 for details): (i) a no-interference Poisson process (Haldane 1919), (ii) a count-location model (Sturt 1976, Karlin and Liberman 1978, Karlin and Liberman 1979, Risch and Lange 1979) accounting for an obligate chiasma (Fledel-Alon et al. 2009), and (iii) a stationary gamma process (Broman and Weber 2000) accounting for chromosomal interference. After the locations of the crossovers are obtained in Morgans they are converted from genetic distance to physical distance and a meiotic product is selected and transmitted (see Supplementary Appendix S1 for details). The user may provide a map to convert genetic distance to physical distance, or else the relationship between genetic and physical distance will be assumed to be linear across the length of the chromosome. The choice of model and the use of a genetic-physical map can alter the distribution of the lengths of segment identical-by-descent (IBD) (Caballero et al. 2019).

### 2.2 Function call

After creating a “genealogy” object, the gen.simuHaplo function can be called by specifying the following arguments: the “genealogy” object, a vector of proband IDs for whom to simulate haplotypes, a vector of founder IDs to include, the number of simulations, the meiosis model, the meiosis model parameters, the length of the chromosomal segment to be simulated, and other optional arguments (see Supplementary Appendix S1 for more details).

### 2.3 Output

**Figure 1:**
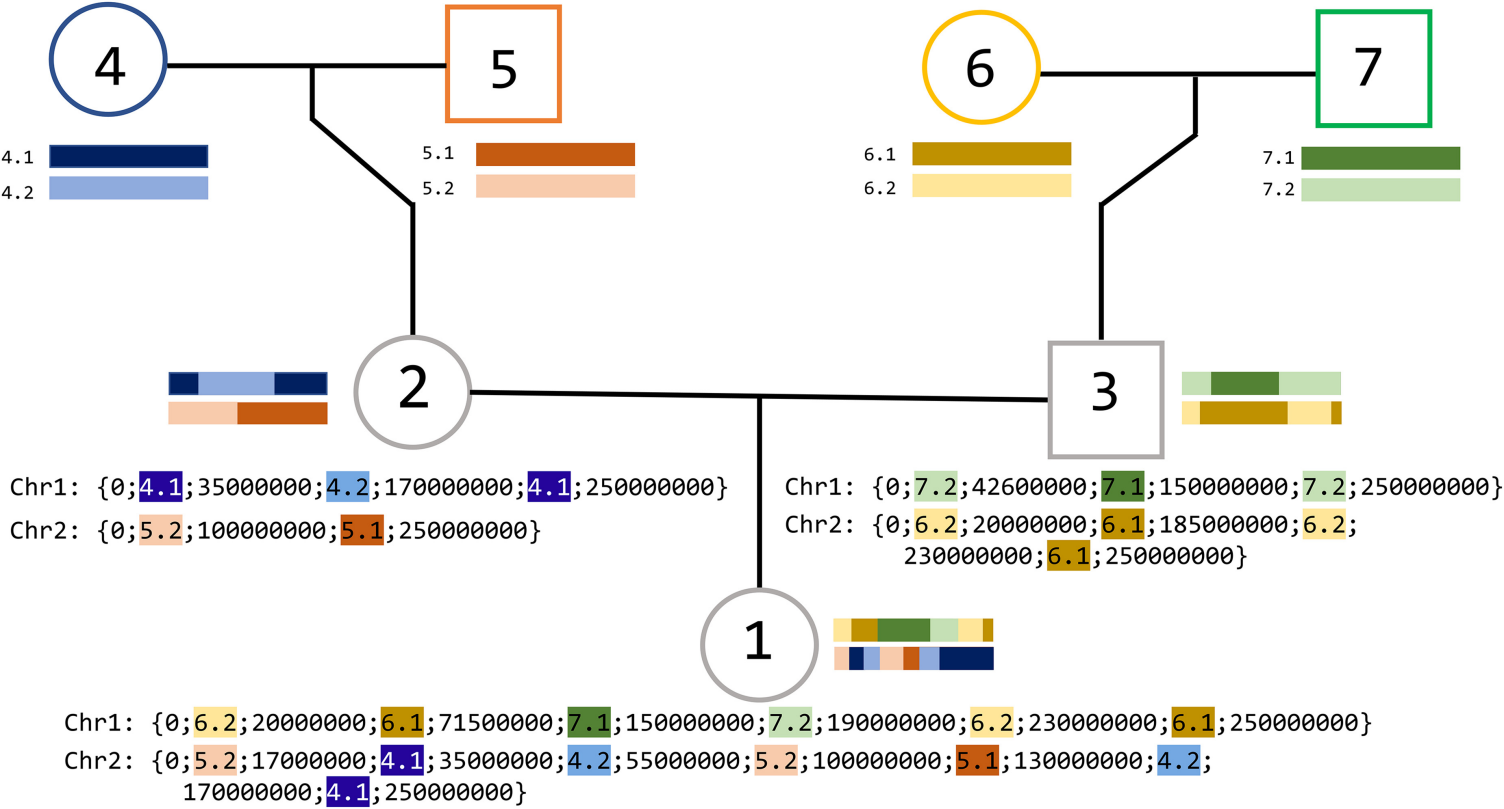
Example simulation of a hypothetical 250 000 000 bp segment. Each individual in the genealogy has a unique integer ID, which is used to label founder chromosomes, e.g.: founder “4” will have chromosomes labeled “4.1” and “4.2”. All founder chromosomes are labeled in this manner. The function iterates through all individuals. For every non-founder individual, we simulate meiosis in both parents and pass down a selected meiotic product from each parent. The notation inside the curly braces demonstrates how the haplotypes appear in the text file output. Segments are identified by their founder of origin (ID #), and the boundary positions are recorded in bp

The output of the gen.simuHaplo function is a text file (Proband_Haplotypes.txt) containing the description of each proband’s simulated haplotypes. An example of the output format is shown in Fig. 1 and more information is provided in Supplementary Appendix S1. Optionally the function can output a second text file (All_nodes_haplotypes.txt) containing the haplotypes for all individuals along the inheritance paths.

### 2.4 Post-simulation functions

Many post-simulation analyses are possible and their types will vary depending on the field of application. The format of the simulation output saved as text files provides maximum flexibility for the different user types. Within GENLIB, we provide two new functions to analyze the simulation output (see Supplementary Appendix S2 for details). The gen.simuHaplo_traceback function retraces genomic segments from probands up to internal ancestors, which can be used, for example, to study the time to coalescence within a large genealogy. The gen.simuHaplo_compare_IBD function can be used to compare the proportion of the diploid chromosome that a pair of probands shares IBD. We show examples using these functions below and in Supplementary Appendix S4.

If users need to simulate with genotype data, we also provide the function gen.simuHaplo_convert for converting the output into genotype data (see Supplementary Appendix S2). The function takes user-provided haploid genotypes for the founders and converts the genomic segments simulated for the probands into corresponding phased genotyped data. Phasing can then be ignored if desired. This could be used, e.g. to distinguish between alleles shared identical by descent versus by state.

## 3 Comparison to other software

Other available gene-dropping software are not designed to efficiently simulate transmission of genomic regions through large genealogies. This is due to software either handling only few loci (making them unable to simulate large regions), tracking a large number of loci (making them inefficient for large genealogies and many replicates), or having additional functionalities (e.g. handling phenotypes). A detailed comparison to other software is provided in Supplementary Appendix S3.

## 4 Examples of applications of gene-dropping simulations

Gene-dropping simulations can be used for many purposes, including estimating the distribution of haplotype lengths in a founder population, the distribution of the length of IBD segments shared by a pair of individuals, or the likelihood of IBD segments being transmitted from ancestors. Simulation results can also be used to compare different statistical methods, as illustrated in Burkett et al. (2022) who used a beta version of gen.simuHaplo (with limited functionalities) to compare genomic- and genealogical/coalescent-based inference of homozygosity by descent in two different pedigree structures from the French-Canadian founder population. Additional examples are presented in Supplementary Appendix S4. We briefly describe one of them below.

The theoretical distribution of the lengths of inherited segments for a given proband–founder relationship is difficult to obtain in consanguineous populations (Nelson et al. 2018). We used the gen.simuHaplo and gen.simuHaplo_traceback functions to estimate the distributions of inherited segments for each possible path of inheritance between a specific founder and a specific proband in a genealogical dataset constructed using the BALSAC database (Vézina and Bournival 2020) from a sample of French-Canadian patients in ophthalmology clinics of Maisonneuve-Rosemont Hospital in Montreal, Canada (Varin et al. 2017, Varin et al. 2020). There were six possible paths of inheritance between the founder and proband (Supplementary Fig. S7). Of 25 000 simulations performed, 4968 resulted in a segment being inherited from the founder, 4961 of which involved a single inheritance path. Seven segments were inherited through a “concatenation” event (i.e. multiple paths from the founder joined at a homozygous internal ancestor). Although rare, these events lead to much longer inherited segments (Supplementary Fig. S8).

## 5 Conclusion

The gen.simuHaplo function combines the GENLIB R package’s existing support for handling large genealogies to allow users to simulate inheritance of large genomic regions with a high density of markers even in genealogies with consanguinity and hundreds of thousands of individuals. To our knowledge, no other simulators with similar functionalities support such large and complex genealogies and marker densities.

## Supplementary Material

btad136_Supplementary_Data

## Data Availability

The data used in the examples shown in this article will be shared on reasonable request to the corresponding author.

## References

[btad136-B1] Bourgain C , Genin E. Complex trait mapping in isolated populations: are specific statistical methods required? Eur J Hum Genet 2005;13:698–706. 15785775 10.1038/sj.ejhg.5201400

[btad136-B2] Broman KW , Weber JL. Characterization of human crossover interference. Am J Hum Genet 2000;66:1911–26. 10801387 10.1086/302923 PMC1378063

[btad136-B3] Burkett KM , Rakesh M, Morris P et al Correspondence between genomic- and genealogical/coalescent-based inference of homozygosity by descent in large French-Canadian genealogies. Front Genet 2022;12:808829. 35126470 10.3389/fgene.2021.808829 PMC8814340

[btad136-B4] Caballero M , Seidman DN, Qiao Y et al Crossover interference and sex-specific genetic maps shape identical by descent sharing in close relatives. PLoS Genet 2019;15:1–29. 10.1371/journal.pgen.1007979 PMC6944377 31860654

[btad136-B5] Charrow J. Ashkenazi Jewish genetic disorders. Fam Cancer 2004;3:201–6. 15516842 10.1007/s10689-004-9545-z

[btad136-B6] Chen H-S , Hutter CM, Mechanic LE et al Genetic simulation tools for post-genome wide association studies of complex diseases. Genet Epidemiol 2015;39:11–9. 25371374 10.1002/gepi.21870 PMC4270837

[btad136-B7] Cheng H , Garrick D, Fernando R. XSim: simulation of descendants from ancestors with sequence data. G3 (Bethesda) 2015;5:1415–7. 25953958 10.1534/g3.115.016683 PMC4502375

[btad136-B8] Chong JX , Ouwenga R, Anderson RL et al A population-based study of autosomal-recessive disease-causing mutations in a founder population. Am J Hum Genet 2012;91:608–20. 22981120 10.1016/j.ajhg.2012.08.007 PMC3484657

[btad136-B9] Falchi M , Forabosco P, Mocci E et al A genomewide search using an original pairwise sampling approach for large genealogies identifies a new locus for total and low-density lipoprotein cholesterol in two genetically differentiated isolates of Sardinia. Am J Hum Genet 2004;75:1015–31. 15478097 10.1086/426155 PMC1182138

[btad136-B10] Fledel-Alon A , Wilson DJ, Broman K et al Broad-scale recombination patterns underlying proper disjunction in humans. PLoS Genet 2009;5:1–7. 10.1371/journal.pgen.1000658 PMC2734982 19763175

[btad136-B11] Gauvin H , Lefebvre J-F, Moreau C et al GENLIB: an R package for the analysis of genealogical data. BMC Bioinformatics 2015;16:160. 25971991 10.1186/s12859-015-0581-5 PMC4431039

[btad136-B12] Haldane JBS. The combination of linkage values, and the calculation of distances between the loci of linked factors. J Genet 1919;8:299–309.

[btad136-B13] Heyer E. One founder/one gene hypothesis in a new expanding population: Saguenay (Quebec, Canada). Hum Biol 1999;71:99–109. 9972101

[btad136-B14] Karlin S , Liberman U. Classifications and comparisons of multilocus recombination distributions. Proc Natl Acad Sci USA 1978;75:6332–6. 16592601 10.1073/pnas.75.12.6332 PMC393176

[btad136-B15] Karlin S , Liberman U. A natural class of multilocus recombination processes and related measures of crossover interference. Adv Appl Probab 1979;11:479–501.

[btad136-B16] Libiger O , Schork NJ. A simulation-based analysis of chromosome segment sharing among a group of arbitrarily related individuals. Eur J Hum Genet 2007;15:1260–8. 17700628 10.1038/sj.ejhg.5201910

[btad136-B17] Liu F , Arias-Vásquez A, Sleegers K et al A genomewide screen for late-onset Alzheimer disease in a genetically isolated Dutch population. Am J Hum Genet 2007;81:17–31. 17564960 10.1086/518720 PMC1950931

[btad136-B18] Maccluer JW , Vandeberg JL, Read B et al. Pedigree analysis by computer-simulation. Zoo Biol 1986;5:147–60.

[btad136-B19] Nelson D , Moreau C, de Vriendt M et al Inferring transmission histories of rare alleles in population-scale genealogies. Am J Hum Genet 2018;103:893–906. 30526866 10.1016/j.ajhg.2018.10.017 PMC6288464

[btad136-B20] Norio R. The Finnish disease heritage III: the individual diseases. Hum Genet 2003;112:470–526. 12627297 10.1007/s00439-002-0877-1

[btad136-B21] Ober C , Abney M, McPeek MS. The genetic dissection of complex traits in a founder population. Am J Hum Genet 2001;69:1068–79. 11590547 10.1086/324025 PMC1274354

[btad136-B22] Pastinen T , Perola M, Ignatius J et al Dissecting a population genome for targeted screening of disease mutations. Hum Mol Genet 2001;10:2961–72. 11751678 10.1093/hmg/10.26.2961

[btad136-B23] R Core Team. R: A Language and Environment for Statistical Computing. 2021.

[btad136-B24] Risch N , Lange K. An alternative model of recombination and interference. Ann Hum Genet 1979;43:61–70. 496395 10.1111/j.1469-1809.1979.tb01549.x

[btad136-B25] Sturt E. A mapping function for human chromosomes. Ann Hum Genet 1976;40:147–63. 1015810 10.1111/j.1469-1809.1976.tb00175.x

[btad136-B26] Varin M , Kergoat M-J, Belleville S et al Age-related eye disease and participation in cognitive activities. Sci Rep 2017;7:17980. 29269882 10.1038/s41598-017-18419-2 PMC5740122

[btad136-B27] Varin M , Kergoat M-J, Belleville S et al Age-related eye disease and cognitive function: the search for mediators. Ophthalmology 2020;127:660–6. 31727427 10.1016/j.ophtha.2019.10.004

[btad136-B28] Vézina H , Bournival JS. An overview of the BALSAC database: past developments, current state and future prospects. Hist Life Course Stud 2020;11:1–17.

