# Supplementary material for: GENLIB: new function to simulate haplotype transmission in large complex genealogies
Source: Bioinformatics. 2023 Mar 17;39(3):btad136. doi: 10.1093/bioinformatics/btad136 (PMC10049784; doi:10.1093/bioinformatics/btad136)
Supplement: btad136_Supplementary_Data [file btad136_supplementary_data.docx]

**Supplementary Material**

**Appendix 1: Details on implementation, usage and output**

**1.1 Implementation**

Most of GENLIB (including the gen.simuHaplo function) is written in C++ and uses the Rcpp package (Eddelbuettel and Francois, 2011), as well as the R core C API to interface with the R environment. The main input for the new gen.simuHaplo function is a “genealogy” object created by the gen.genealogy function. This object contains the following information pertaining to each individual in the genealogy: identification number (ID), father ID, mother ID and sex. The gen.simuHaplo first creates a list of individuals from the “genealogy” object such that any dependent individual is listed after their ancestors (i.e.: parents before children, and all ancestors before descendants). Since a family tree is a directed acyclic graph, we can always perform such a topological sort. Then we iterate through the list, and, for each individual, we simulate meiosis in the parents according to one of three possible models described below and pass down the meiotic products.

A meiotic product is created by first obtaining a list of chiasma positions (in base pairs, BP), then assuming that each chiasma has a probability of 0.5 of appearing in the given meiotic product as a crossover. After selecting all the crossovers, we select the parental copy with which the meiotic product begins with probability of 0.5. We copy over the parental chromosome until reaching a crossover position, then we alternate to copying from the other parental chromosome. The chromosomes are stored as linked lists where each segment contains its end position in BP, and points to the next segment in the chromosome.

## Model 1: Poisson process (Haldane, 1919)

If the user specifies “model=1” then a Poisson process will be used to simulate meiosis. The “model_params” argument of the function should be used to pass in a 2-element vector specifying the sex-specific recombination rates (in Morgans) of the Poisson process. For this model, the “model_params” argument should be the same as the “genetic_length” argument. This is because we use a Poisson process with rate L (length of region to be simulated in Morgans) to simulate the positions of the crossovers. Since a thinned Poisson process is also a Poisson process, we simulate the crossovers directly i.e., using a crossover process with rate L, instead of a chiasma process with rate 2L.

The number of crossovers is generated by sampling from a Poisson distribution with rate L, the sampled number of crossovers are then uniformly randomly distributed over the length of the region (in genetic distance). The crossovers positions are then converted from genetic distance into physical distance. Once we have the physical locations of the crossovers, a meiotic product is created to pass down to the offspring.

## Model 2: Zero-Truncated Poisson Count-Location model (Karlin and Liberman, 1978; Karlin and Liberman, 1979; Risch and Lange, 1979; Sturt, 1976).

If the user specifies “model=2” then a zero-truncated Poisson (ZTP) distribution will be used to generate the number of chiasmata:

$$p_{n}=\frac{e^{-\lambda}\lambda^{n}}{n!(1-e^{-\lambda})}, n>0$$

After obtaining the number of chiasmata, they will be distributed randomly along the simulated region, then the chiasma will be selected with p=0.5 to obtain the crossover positions. Note that this model guarantees a chiasma (obligate chiasma), but there still may be 0 observed crossovers.

The user must specify the parameter $\lambda$ of the ZTP distribution using the “model_params” argument. The user must pass in a 2-element vector to specify a parameter for each sex, which can be the same. One way to select an appropriate value of $\lambda$ would be to use the following equation and set E[X] to 2L, where L is the length of the simulated region in Morgans (we use 2L since we are modelling chiasmata):

$$E[X]=\frac{{\lambda e}^{\lambda}}{e^{\lambda}-1}$$

To sample from this distribution, we sample from a standard Poisson distribution with the specified parameter and resample if we obtain a value of 0.

## Model 3: Stationary Gamma process (Broman and Weber, 2000)

If the user specifies “model=3” then a stationary Gamma renewal process as described in Broman and Weber (2000) will be used to simulate meiosis. The Gamma renewal process models the “interarrival” distances between chiasma using a Gamma(v, 2v) distribution, where v is the shape parameter, and 2v is the rate parameter. This restriction of 2v as the rate parameter keeps the expected value of the distribution to 1/2, for an average distance of 0.5 Morgans between chiasmata. The user must specify the sex-specific values of v using the “model_params” argument.

The distance to the first chiasma is distributed differently than the rest of the inter-chiasma distances, which are distributed Gamma(v,2v). We obtain the distribution of the distance until the first chiasma (first arrival time) by selecting a distribution that will satisfy the stationary property. The stationary property guarantees that the probability of chiasma formation will be equal anywhere along the chromosome, and the p and q terminals of the chromosome will be treated the same regardless which terminal we initiate the process at.

If the first arrival time is distributed according to the limiting distribution ${\mathcal{F}(x)}/\mu$of the interarrival distribution, then the renewal process will have the stationary property. Here $\mathcal{F}(x)$ is the survival function of Gamma(v,2v), and $\mu$ is the mean (1/2). This is equivalent to 2[1-F(x)] where F(x) is the cumulative distribution function (CDF) of Gamma(v,2v). We evaluate the CDF using source code obtained from (https://people.math.sc.edu/Burkardt/cpp_src/asa239/asa239.html), which uses the algorithm for calculating the incomplete Gamma integral from (Shea, 1988). We generate 10,000 partial Riemann sums for 2[1-F(x)] for x values between 0 and the length of the region (in Morgans). Then we sample from the 2[1-F(x)] using an inverse transform method using the partial sums.

After sampling the position of the first chiasma, we then sample from the Gamma(v,2v) distribution (implemented in C++ std random library) to get the distance to the next chiasma. We continue this until the length of the region (in Morgans) is exceeded. We then independently select crossovers from the chiasmata, and convert the positions into BP, same as the other two models.

For all the models of meiosis, we use the C++ std random library for the Mersenne twister pseudorandom number generator, and the random distributions (Poisson, Uniform, Gamma).

## Using a map to convert genetic distance into BP

Genetic distance does not perfectly correlate with physical distance along the simulated region. For instance, there are fewer observed crossovers around the centromere. In addition, the distribution of recombination sites seems to be different for males and females as well, e.g.: males have been observed to have higher rate of recombination around the telomeres (Bherer, et al., 2017). As discussed in Caballero et al. (2019), using maps to convert genetic distance into physical distance as part of simulating meiosis can alter the distributions of identity-by-descent (IBD) sharing between pairs of individuals of a given relatedness (Caballero, et al., 2019).

To specify a map to the gen.simuHaplo function, the map must be a data frame with a column named “cM” and a column named “BP”. Each row of the data frame should be an ordered pair that identify a point on the map, and the rows should be ordered from low to high (start to end of the simulated region).

When converting a crossover position from genetic distance to physical distance using the map, we find the pair of points that bound the crossover position and use linear interpolation of the physical distances of the points to obtain the physical position of the crossover. This can be an expensive procedure if the map consists of many points (high resolution map) since we have to search this map for every meiosis. We recommend approximating the map with a few points as shown in Supplementary Figure 1.

**
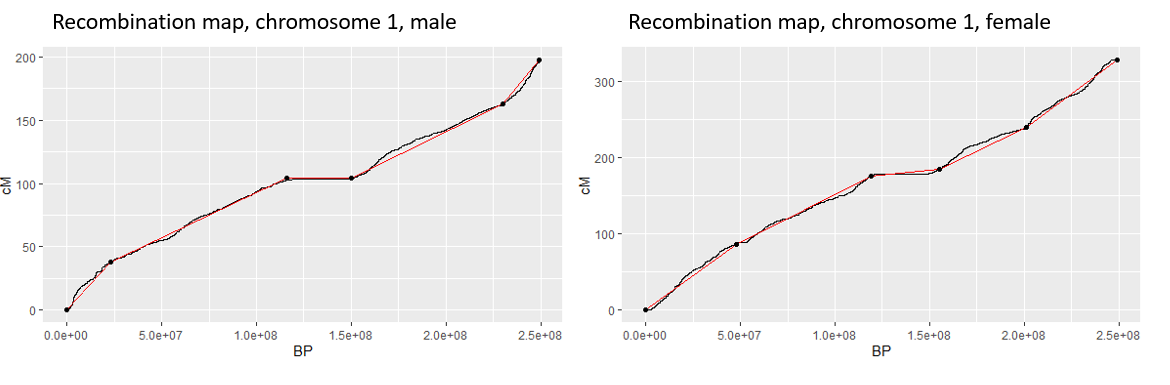
1.2 Usage**

**Supplementary Figure 1.** High-resolution chromosomal maps (black lines) from Bherer et al., (2017) constructed from 100,000 + recombinations sampled from family studies. The red line shows a potential piece-wise approximation that would be more efficiently used by the simulation function.

The gen.genealogy function takes a genealogy table input and converts it into an object usable by the GENLIB functions. The input genealogy table must be a matrix or data frame with the following columns: “ind”, “mother”, “father”, and an optional “sex” column. Each individual in the genealogy should be represented by a row in the table, with a unique integer ID in the ind column. Individuals missing a mother and/or father in the genealogy should have the mother and/or father ID numbers set to 0. Sex can be specified as M or 1 for males and F or 2 for females. The sex column is optional, and not necessary for gen.simulHaplo, but necessary for some other functions using the “genealogy” object. For gen.simulHaplo all founders must be present as an individual in the genealogy, i.e., the founders have an ID and their parents’ IDs are coded as ID=0. The gen.genealogy function has an autoComplete option, which when set to TRUE, will automatically complete the genealogy (any ID numbers in the mother/father columns that are not in the individual column will be added with mother/father set to 0).

The gen.simuHaplo function takes the following arguments:

- “gen”—The “genealogy” object, created using gen.genealogy. Required.
- “pro”—A vector of proband IDs for whom to simulate haplotypes (defaults to all individuals without children in the input genealogy).
- “ancestors”—A vector of founder IDs to include (defaults to all founders of the specified probands). The founders of the specified probands who are not included in this vector will have their chromosome labelled as 0.
- “simulNo”—The number of simulations to run, defaults to 1.
- “model”—An integer value (1,2, or 3) to specify the model of meiosis to use. Defaults to 1 (Poisson process).
- “model_params”—The parameter for the model of meiosis. Vector of length 2 to allow for sex-specific parameters (first element is male, second for females). Required.
- “cM_len”—The genetic length of the chromosome or chromosomal segment (in centiMorgans) passed as a vector of two values (cM length for males, cM length for females). Required.
- “BP_len”—The physical length of the chromosome in BP (integer value). Required.
- “physical_map_male”—An optional map to specify the relationship between genetic length and BP location. A dataframe with the required columns named “BP” and “cM”. Each row in this dataframe corresponds to a point along the chromosome in terms of both genetic and physical length, and the map is built piecewise from these points. The first row should be (0,0) (the start of the chromosome), and the last row should be the length of the chromosome in cM, and BP. If this map is not specified, then the chromosome is assumed to be linear in terms of the physical-to-genetic distance relationship.
- “physical_map_female”—Same as above, but for females (sex-specific maps).
- “seed”—An optional integer seed value for the random number generator.
- “all_nodes”—An indicator for the output of an additional file containing the haplotypes of all the internal individuals of the genealogy (not just the probands).
- “outDir”—An optional string specifying an output directory (defaults to the current working directory).

More details on usage can be found in “example 1” on the GENLIB GitHub (<https://github.com/R-GENLIB/simuhaplo_functions>).

**1.3 Output**

The first line of the “Proband_Haplotypes.txt” file shows the number of simulations and the number of probands. For example, “100;227” means that 100 simulations were performed on 227 probands. The rest of the file includes one line for every proband for every simulation. Each line contains haplotype information in a set of 3 curly braces (see Application Note Figure 1). In the first curly brackets are the simulation number, the proband ID, and a placeholder spot for future developments of the function. The next two sets of brackets contain the haplotype information for the diploid chromosome. The first of these two is the paternal chromosome copy, and the second is the maternal copy.

The haplotypes are encoded {position ; origin_ID; position; origin_ID; … origin_ID; position} where the positions are BP positions along the chromosome, and origin_ID refers to the ID number of the founder from which the segment between those positions originated. The origin_ID are founder ID numbers with “.1” or “.2” appended to them, to distinguish between the diploid copies of the founder chromosome.

The “All_nodes_haplotypes.txt” file is formatted in a similar manner, with three curly braces per line. The difference is that it has a line for each individual in the simulation, not just the probands, and additionally the first set of curly braces contains more information recording the details for each meiosis (number of crossovers, the position of crossovers, and the inherited meiotic product). These extra details can be used to reconstruct paths of inheritance. More details on output can be found in “example 1” on the GENLIB GitHub (<https://github.com/R-GENLIB/simuhaplo_functions>).

# **Appendix 2: Post-simulation functions**

Two additional GENLIB functions described below can be used to analyze the output of the simulation function: gen.simulHaplo_traceback, and gen.simulHaplo_IBD_compare. Additionally, the function gen.simulHaplo_convert can be used to convert the simulation results to genotype data. Examples on how to use the functions can be found at: <https://github.com/R-GENLIB/simuhaplo_functions> and results from these functions are shown in Appendix 4.

## Tracing segments up the transmission path

The gen.simulHaplo_traceback function can be used for tracing back a segment in a proband’s simulated haplotype up the transmission path by identifying the boundaries of the segment to be traced back, and then following the recombination history stored in the “All_nodes_haplotypes.txt” output file upwards at each meiosis until reaching the founder.

The gen.simulHaplo_traceback function takes the following arguments: the “genealogy” object that was used for the simulation, the ID of a proband, and the ID of the founder, the path to the “All_nodes_haplotypes.txt” file and the path to the “Proband_Haplotypes.txt” file. Any segment originating from the specified founder that is present in the specified proband’s simulated haplotype will be traced back up the inheritance path. The traceback function lets us check the exact path followed for the inheritance of a specific segment, in each simulation. This information could be useful for identifying internal ancestors who play an important role in the inheritance of mutations under study, or to study the time to coalescence of a segment within a large genealogy. The output file “All_nodes_haplotypes.txt” can get very large, so it is prohibitive to output this file when performing millions of replicates in large genealogies. Planned future GENLIB updates include the option of outputting compressed binary files to support traceback in millions of simulations in large genealogies.

The “All_nodes_haplotypes.txt” file contains the details for each meiosis (locations of crossovers and which meiotic product was passed on), so to trace a segment up its inheritance path the function checks the number of crossovers that occurred before the start of the segment, and identifies which parent that segment was inherited from, and then iterates until reaching the founder.

The traceback function returns a data frame that contains three columns: “simulNo’, “seg_length”, and “path_n”. Each row in the data frame corresponds to a segment present in the proband haplotype that is inherited from the founder. The “simulNo” column specifies the simulation number, the “seg_length” column is the length of the inherited segment, and the “path_n” column specifies an integer key that corresponds to the specific path of inheritance for the segment. If in any of the simulations the proband has inherited multiple discontinuous segments from the founder then there will be multiple consecutive rows created all with the same “simulNo” identifier. Additionally, the function will print to the R console all the paths of inheritance (the path is represented by the series of individual ID numbers for all individuals in the path) that were observed in the simulations. The paths printed to the R console are numbered, and the number corresponds to the identifier in the “path_n” column.

## IBD sharing between pairs of probands

The gen.simulHaplo_IBD_compare function is used to compare the proportion of the diploid chromosome that a pair of probands share IBD. The function takes the following arguments: “proID_1”—the integer ID number of the first proband, “proID_2”—the integer ID number of the second proband, “BP_len”—the length of the simulated chromosome in BP, and “proband_haplotypes_path”—a string with the path to the “Proband_Haplotypes.txt” output file.

The gen.simuHaplo function simulates both copies of a diploid chromosome for every individual, so when determining the percent shared IBD between a pair of individuals we must compare both copies to each other. Since we have a numerical identifier for the origin of each segment we check the diploid haplotypes for the pair of individuals for any segments with the same ID, at overlapping positions. Once all overlapping regions are determined, we need to check if any of the identified IBD segments are directly adjacent to each other (i.e.: a longer IBD stretch inherited from a common internal ancestor, that is composed of multiple segments from different founders), then we consider it as one long segment. This function implicitly treats each founder chromosome as completely unrelated since only segments with the same ID will be counted IBD.

When comparing the two individuals of a pair, both copies of the chromosome for individual 1 must be compared to both copies of the chromosome for individual 2. For instance, when determining the IBD regions for individual 1 chromosome copy 1, we must compare it with both copies from individual 2. Additionally, if an individual is homozygous by descent (HBD) in one of these IBD regions, then we must avoid double counting the HBD part of the IBD regions when reporting the total IBD for the pair. For example, if individual 1 chromosome copy 1 has a segment IBD with both of individual 2’s chromosomes (which are HBD for some IBD stretch) then we want to avoid double counting the HBD region when calculating the total IBD proportion for the pair. This region should be fully counted when tallying individual 2’s IBD sharing length, but when tallying individual 1’s IBD sharing we should not double count the overlapping HBD portion.

After identifying the IBD regions for each of the 4 chromosomes the function then returns a dataframe with the following four columns: “simulNo”—the simulation number under consideration (will only return simulations with non-zero IBD sharing between the pair); “n_seg”—the number of discontinuous segments shared between the pair (note: this is for the pair so divide by 2 to get an “average”), this number can sometimes be odd if an individual is HBD over one of the IBD regions; “pIBD”—the proportion of the diploid chromosome shared IBD; and “mean_seg_len”—the average length of all the IBD segments. Additionally, the function will print the position and length of every IBD segment to the R console, which can be supressed by disabling messages in R.

Convert genomic transmission information to genotype data

The gen.simuHaplo function does not return genotype data. We provide the GENLIB function gen.simulHaplo_convert a function to convert the simulated genomic segments obtained from gen.simuHaplo to genotype data for the probands using a file of user-provided founder haploid genotypes. This function replaces the simulated segments with appropriate stretches of sequence data from the genotype data for the respective founder chromosomes (using the BP positions of the segment).

The founder genotype information should be contained in two files, named “founders.hap” and “founders.map”. The “founders.hap” file should be formatted such that each line starts with the numerical identifier for the founder chromosome, followed by a whitespace, and then a string specifying the genotype. Haploid genotypes may be described as a string of any characters, so long as each founder chromosome has a string of the same length. Each single character in the string represents the genotype at a specific BP position. The missing data should be included (encoded with any single character) so that all the sequences are same length. The “founders.map” file should contain the BP position of each character (variant genotyped); the file should have the same number of lines as there are characters in the haploid genotype string, with only a single integer on each line (BP position). An example can be found at the GitHub link above, with sample data that shows the file formatting.

The gen.simuHaplo_convert function only takes one argument, a path to a directory where it expects to find the three files “Proband_Haplotypes.txt”, “founders.hap”, and “founders.map”, by default this will be set to the working directory. After running the function, a new file “Proband_Genotypes.txt” is generated in the directory.

# **Appendix 3: Comparison to other software**

Use of simulation tools is widespread throughout genetic epidemiology, human and population genetics, animal and plant breeding. Simulation approaches can be considered as either backward-in-time, or forward-in-time. Forward simulators are used to simulate the progression of a population under some models of fitness, selection, mating, population size, mutation rates etc. Many tools do not let users specify a pedigree since they are meant to simulate the mating. Most of the gene-dropping tools that follow user-specified pedigrees are created to track specific alleles, evaluate phenotypes, or condition on observed data (Li, et al., 2015; Nieuwoudt, et al., 2020).

Libiger and Schork (2007) published a gene-dropping tool to investigate IBD segment sharing. Their approach tracks states of particular marker alleles as opposed to the positions of segments. We could not locate software corresponding to the paper. SimPed (Leal et al., 2005) is another such software that performs gene-dropping on individual marker loci. The software was obtained from (<https://www.hgsc.bcm.edu/software/simped>) and we attempted to use it to simulate 25 hypothetical markers, each spaced 0.1 Morgan (10 centimorgan, cM) apart for our Montreal genealogy. The software had a hard-coded maximum of 3000 individuals per genealogy. After altering this and recompiling the source code, the program crashed while attempting to simulate results for our genealogy. On small sample genealogies with the same parameters the software worked as intended.

The MORGAN software (Thompson, 2011) package contains a gene dropping program that also tracks the states of marker loci. We tested the ‘genedrop’ tool in Morgan v3.4 using the same approach of 25 hypothetical markers (all with allele frequency 0.5), each spaced 10cM apart. For a single simulation Morgan was able to generate the results in 1.95sec (on a 2.9GHz CPU machine). When increased to 100 markers each spaced 2.5cM apart a single simulation took 7.17sec, and finally for 1000 markers spaced 0.25cM apart the simulation took 58.0sec. However, for a test of 10000 markers spaced 0.025cM apart the program returned an error, so it seems genedrop is not appropriate for simulating high-density marker data. On the same machine GENLIB took 0.83sec on average to run one simulation.

Since our tool only tracks the position and origin of chromosomal segments it is more lightweight and can efficiently simulate transmission in very large, complex genealogies. In addition, the gen.simulHaplo_convert function can be used to convert the output to marker/sequence data as described above. Since this function just copy-pastes the founder sequence data into the appropriate order, it is fast for any number of markers used and does not impact simulation time since the conversion is performed after the simulation. Hence, GENLIB is more apt for running many simulations and using the results to investigate patterns of inheritance, which is why GENLIB provides the option of specifying genetic maps, different models of meiosis, and allowing different parameters for males and females.

XSim, as described in Cheng (2015) uses the approach of tracking positions and origins of chromosomal segments, however the software (<https://github.com/reworkhow/XSim.jl>) is designed for simulating breeding schemes, and mating programs, and not for dropping down user defined pedigrees.

The available software most similar to ours are PedSIM (Caballero, 2019) and IBDsim (<https://cran.r-project.org/web/packages/IBDsim/index.html>). However, both tools are designed for the evaluation of close relatives (small pedigrees).

PedSim has a unique file format specification for the input pedigrees. The file format as described at (<https://github.com/williamslab/ped-sim#def-fileigree simulator>) is not amenable for genealogies with consanguineous loops, or individuals that appear in multiple generations. The script provided in the github repository to convert PLINK “*.fam” file format to the PedSim file format fails to convert our example genealogy.

IBDsim, which is part of the Pedsuite (<https://cran.r-project.org/web/packages/pedsuite/index.html>) set of tools crashes when using the ped() command to try to load in our example pedigrees.

# **Appendix 4: Example Applications**

Here we use our function to simulate haplotypes for probands in two genealogies. We illustrate some of the analyses that can be performed. We explore two genealogical structures of over 15 generations from the French-Canadian founder population, both constructed using the BALSAC database (BALSAC project, Université du Québec à Chicoutimi, https://balsac.uqac.ca/) (Vézina and Bournival, 2020). The first genealogy was constructed from a sample of individuals recruited in a family study of asthma in the Saguenay-Lac-Saint-Jean (SLSJ) region (Laprise, 2014), which harbors higher levels of inbreeding due to its founding history and isolation. The second genealogy was constructed from a more outbred sample from patients in ophthalmology clinics of Maisonneuve-Rosemont Hospital in Montreal (Varin, et al., 2017; Varin, et al., 2020). More details on the genealogies used in the example applications can be found in Burkett, et al., (2022). Subsets of the present-day individuals were selected from the two genealogies to include in our simulations, yielding the genealogies shown in Supplementary Table 1. We simulate the equivalent of chromosome 1, with a length of 290,000,000 BP and genetic length of 198cM in males, and 328 cM in females.

Supplementary Table 1. Overview of the two genealogies used for simulations

|  | SLSJ | Montreal |
| --- | --- | --- |
| Number of probands included | 226 | 227 |
| Number of founders | 7608 | 9095 |
| Number of individuals | 55750 | 58942 |

## Comparing the models of meiosis

To compare how the models of meiosis can affect the haplotype inheritance among probands we run 1000 simulations for each model of meiosis, both with and without the use of genetic-physical map. For the Poisson model we use 1.98, and 3.28 as our model parameters (length of chromosome in Morgans), for the ZTP model the parameters are 3.88, and 6.55, which we obtain from the equation in Appendix 1 to keep the same expected number of crossover, and for the Gamma model we use 4.5 and 5.5 as our parameters, which were the values found by (Broman and Weber, 2000) after fitting the Gamma model to their observed recombination data. The gamma model will always have expected number of crossovers equal to the Morgan length, so all 3 models have same expected number of recombinations per meiosis. The maps used are the approximations shown in Supplementary Figure 1.

Run times for the different meiosis models are shown in Supplementary Table 2. Using a more complicated Gamma model with a map adds ~1 minute to the simulation time compared to a simple Poisson model with no map.

Supplementary Table 2: run time, and output file size for 1000 simulations in both genealogies. Simulation was run on a i3-4160 CPU @ 3.60GHz machine. The function is currently only single thread.

|  | SLSJ Genealogy | | Montreal Genealogy | |
| --- | --- | --- | --- | --- |
|  | Time (min) | Output file size (Mb) | Time (min) | Output file size (Mb) |
| Poisson no map | 2.96 | 232 | 2.48 | 201 |
| Poisson w/ map | 3.08 | 230 | 2.70 | 200 |
| ZTP no map | 3.03 | 231 | 3.00 | 201 |
| ZTP w/ map | 3.23 | 230 | 3.02 | 200 |
| Gamma no map | 3.63 | 231 | 3.46 | 201 |
| Gamma w/ map | 3.75 | 230 | 3.56 | 200 |

We first compare the distributions of longest inherited founder segments between the different models of meiosis. For each of the 1000 simulation replicates, we obtain the length of the longest inherited founder segment for each proband over both copies of chromosome from all founders, yielding 227,000 segments for the Montreal genealogy and 226,000 segments for the SLSJ genealogy. The distributions are shown in Supplementary Figure 2. The ZTP and Poisson have almost identical distribution, which is to be expected since we are simulating a long chromosome where the chance of sampling 0 chiasma is low. The models that use the map have lower mean and more concentrated distribution. One explanation for this might be because the map sequesters crossovers into the tail regions of the chromosomes, and away from the centromere, this could lead to shorter segments after successive meioses. The gamma models also appear to have less dispersion and be more centered around the mean. This makes sense because the purpose of the gamma model is to account for chromosomal interference, i.e.: to space out recombination events, which would reduce the variance in segment length.

Next, we compare how the different models of meiosis may affect the proportion of IBD sharing. We check every pair of probands (25425 for SLSJ, 25651 for Montreal) to determine whether they share any part of the simulation segment IBD, i.e., we treat sharing as a binary variable. We calculate the frequency of IBD sharing as the proportion of the 1000 simulations where the pair had non-zero levels of IBD sharing. The distributions over all pairs are shown in Supplementary Figure 3. All models have very similar distributions, with the Gamma models showing slightly higher means (horizontal line).


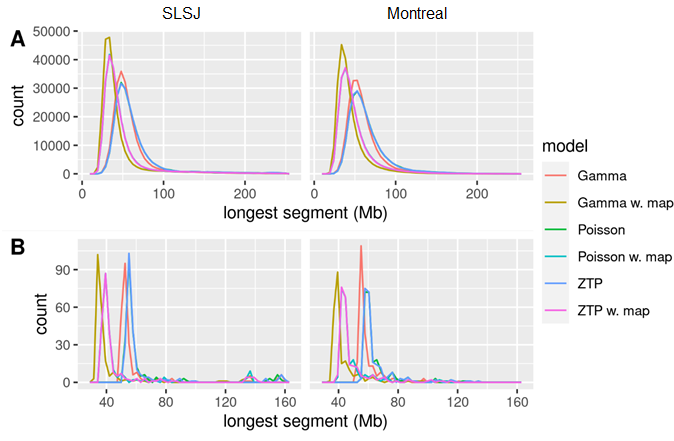
Supplementary Figure 2. A: length of longest segment for all probands, all simulations. B: longest segment, average value for each proband (averaged over simulations).


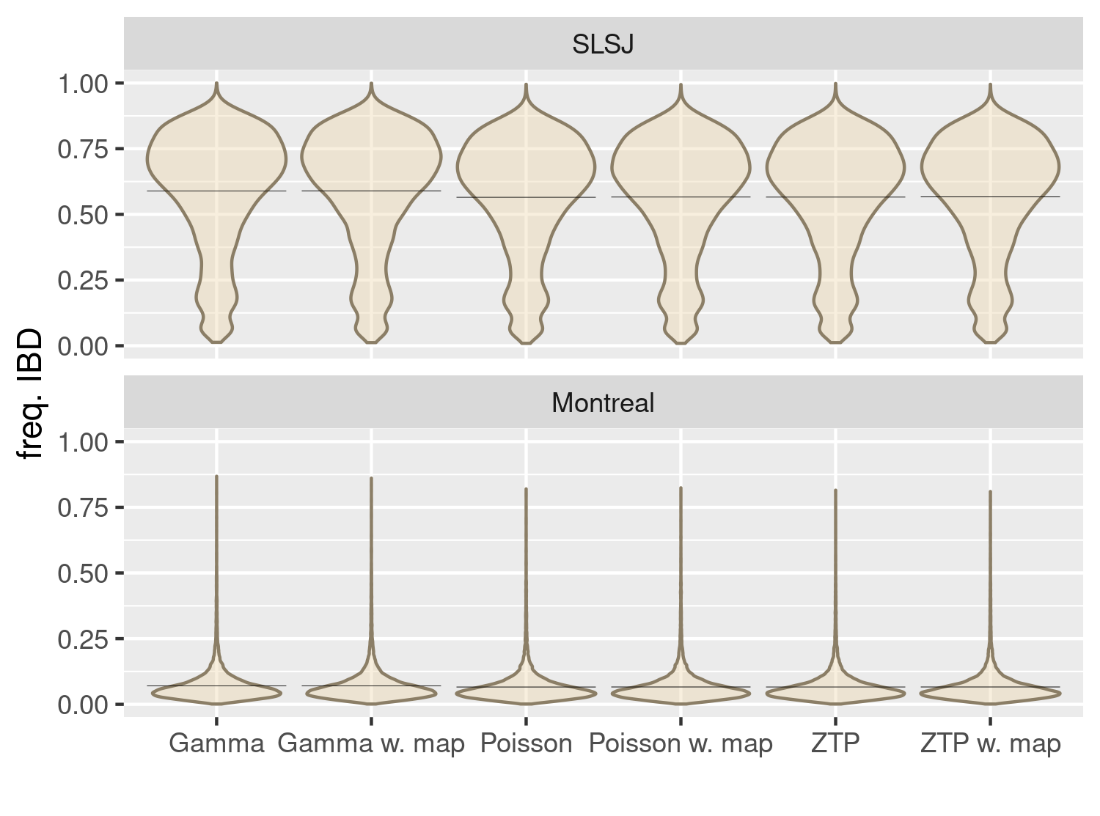
Supplementary Figure 3. Distribution over all pairs (25425 for SLSJ, 25651 for Montreal) of the frequency of IBD sharing (proportion of the 1000 simulations where the pair had non-zero levels of IBD sharing) for the different meiosis models.

## Exploring IBD sharing between pairs of probands

In this example application, we only consider the simulation results from the Poisson model with no map. We check each pair of probands using the gen.simuHaplo_compare_IBD function and obtain details on the IBD sharing between each pair of probands for each of the 1000 simulations. We also obtain a genealogical kinship coefficient between every pair of individuals using the gen.phi command in GENLIB. By definition, the mean of the proportion of the genome shared IBD between a pair should be equal to twice the kinship. We obtain the proportion of the simulated segment shared IBD for each of the 1000 simulations, and compare the mean to the kinship in Supplementary Figures 4 and 5.

As expected, the mean proportion of the simulated segments shared IBD correlates strongly with kinship, with slope 2 and intercept 0, and the variance increases as kinship increases. The patterns of variance can be seen in the zoomed plot (Supplementary Figure 5). In addition, especially in the Montreal genealogy, it seems that the variance is higher, at a given kinship level, for pairs with higher mean proportion of segments shared IBD. This pattern is less apparent in the overall more related SLSJ population.


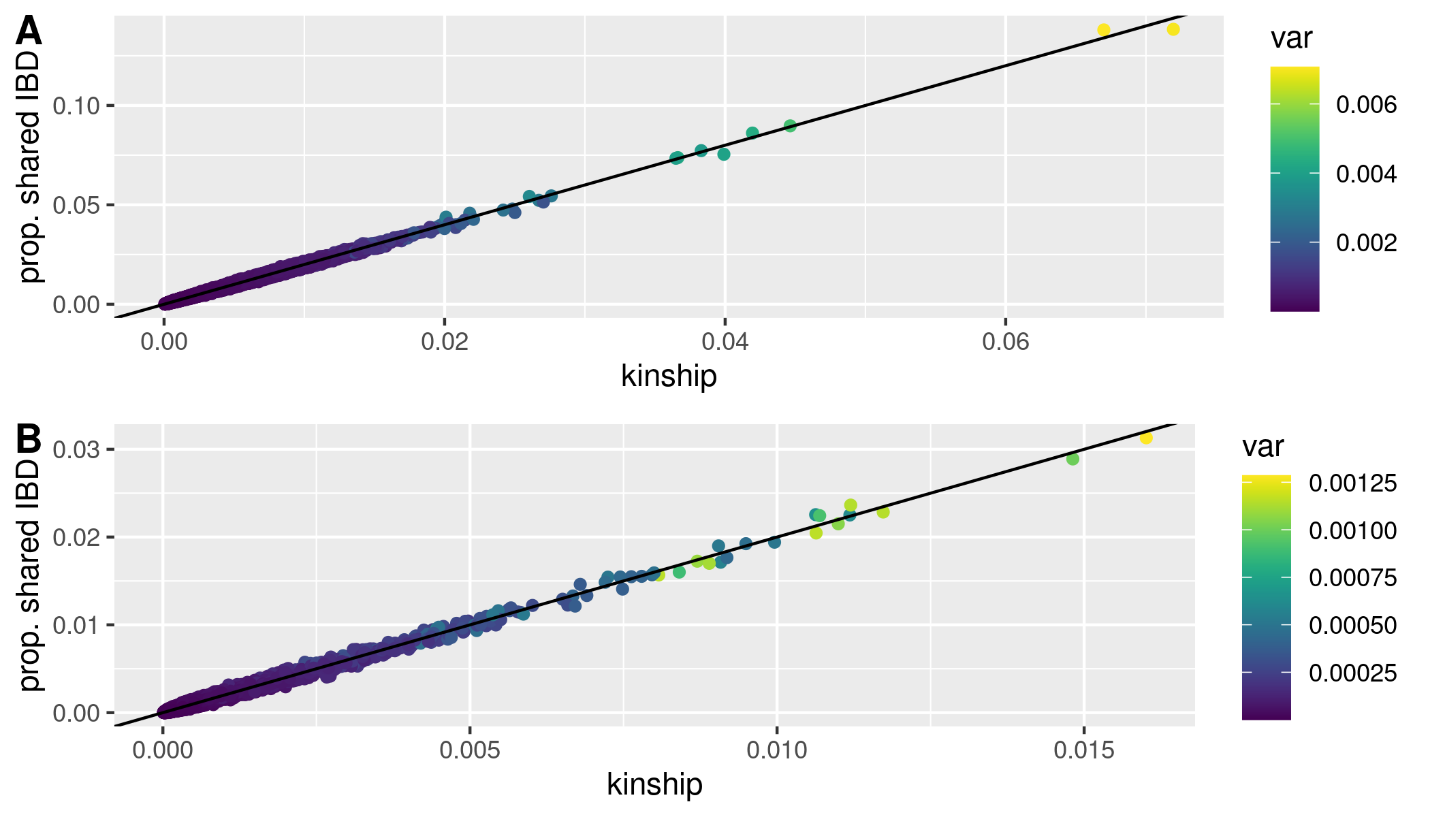


Supplementary Figure 4. Mean proportion of the simulated segment shared IBD vs. genealogical kinship. A: SLSJ genealogy, 25425 proband pairs (226 probands). B: Montreal genealogy, 25651 proband pairs (227 probands). The colours show the variance (var) for IBD sharing over the 1000 simulations. The expected regression line of slope=2 and intercept=0 is shown.


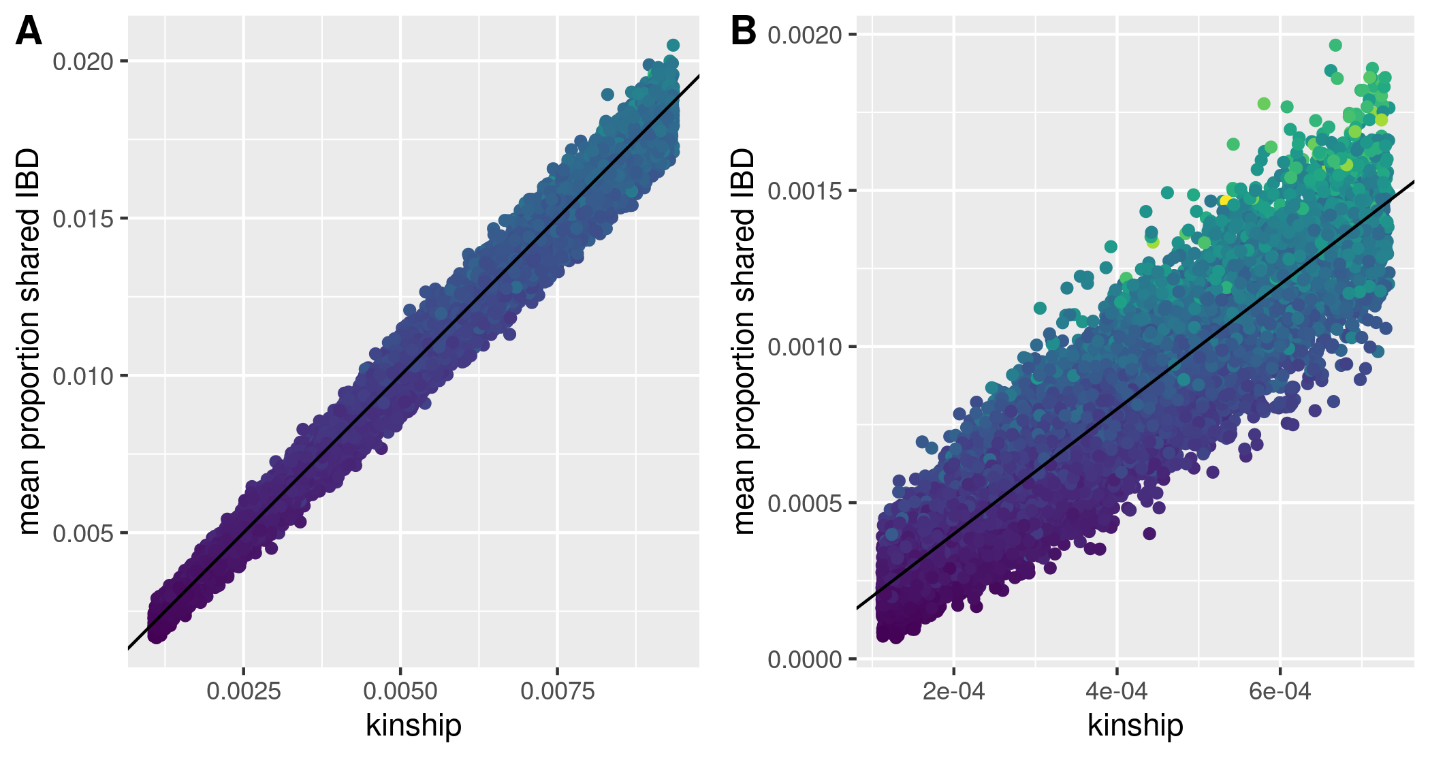


Supplementary Figure 5. Mean proportion of the simulated segment shared IBD vs. Genealogical kinship, zoomed to remove pairs of probands in bottom and top 10 percentile of kinship values. A: SLSJ genealogy, 25425 proband pairs (226 probands). B: Montreal genealogy, 25651 proband pairs (227 probands). The colours show the variance over the 1000 simulations (var legend shown in Figure 4). The expected regression line of slope=2 and intercept=0 is shown.

For a given pair the kinship can only tell us the expected value of the proportion of the genome shared IBD. The distribution can be estimated from the simulation results. A specific value of the kinship coefficient can be realized in different ways, e.g.: a pair of individuals that share a grandparent, and a pair of individuals that share two great-grandparents will have the same kinship coefficient and therefore the same expected value of total percent shared IBD. However, the distributions of the genomic proportion shared IBD may be quite different. To explore this, we selected 4 pairs of probands in the SLSJ genealogy.

Pair 1 and pair 2 have similar kinship values, and the same for pair 3 and pair 4. Both sets of pairs have similar kinship values, but different distribution of IBD sharing (Supplementary Figure 6). Pair 4 and pair 2 realize their kinship earlier in the genealogical history (shown in Fig 6C), meaning their relationship is due to common ancestors at shallower genealogical depth. This explains their higher frequency of no IBD sharing compared to the other pairs. Indeed, when IBD sharing is observed for these pairs, it will likely be on a longer segment but there is a higher probability of sharing no segments at all. In contrast, for pairs 1 and 3, which share common ancestors at a deeper genealogical depth, the length of shared segments is smaller but there is a lower chance of having 0 IBD segments.

## Inheritance of segments from an ancestor

In a theoretical, completely outbred pedigree there would only ever be one path connecting proband to founder. The theoretical length of a segment inherited from this founder would only depend on the number of meioses along that path, and the sex of the individuals along the path (if we consider sex-specific parameters of the meiotic process). One can analytically obtain a distribution for the lengths of inherited segments as described in (Boehnke, 1994) for a given path (assuming a Poisson process model for crossovers), however if there are multiple possible paths as is the case in inbred pedigrees from founder populations, one would have to create a mixture distribution by summing over all possible paths and weight them according to the likelihood of inheritance down a given path.

Additionally, when deriving the distribution for the length of an inherited segment, it is considered that the meioses along the transmission path will potentially shorten the segment (Boehnke, 1994; Caballero, et al., 2019; Nelson, et al., 2018). However, when crossing paths exist it is possible this assumption would be violated. If multiple paths converge at an internal ancestor, it is possible for the internal ancestor to be homozygous for the founder segment, and this ancestor may pass along a haplotype that has been lengthened by a recombination, instead of being shortened. This should be a rare occurrence (depending on the levels of consanguinity), but the gen.simuHaplo_traceback function will be able to identify if it occurs.

Given the difficulty in analytically deriving distributions for lengths of inherited IBD segments for a given proband-founder relationship in consanguineous populations, we can use simulations to estimate this distribution, and using the traceback function, we can further obtain a distribution of inherited segment lengths for each possible path of inheritance.

We ran simulations on a small sub-tree of the Montreal genealogy, we only consider the connections between proband “222” and their ancestor “335” (Supplementary Figure 7). We ran 25,000 simulations (segments not originating from “335” are all given the same identifier of “0”). If “222” inherits a segment from “335” then we can use the traceback function to identify which transmission path was followed. We can also detect any instance of the concatenation event mentioned above.


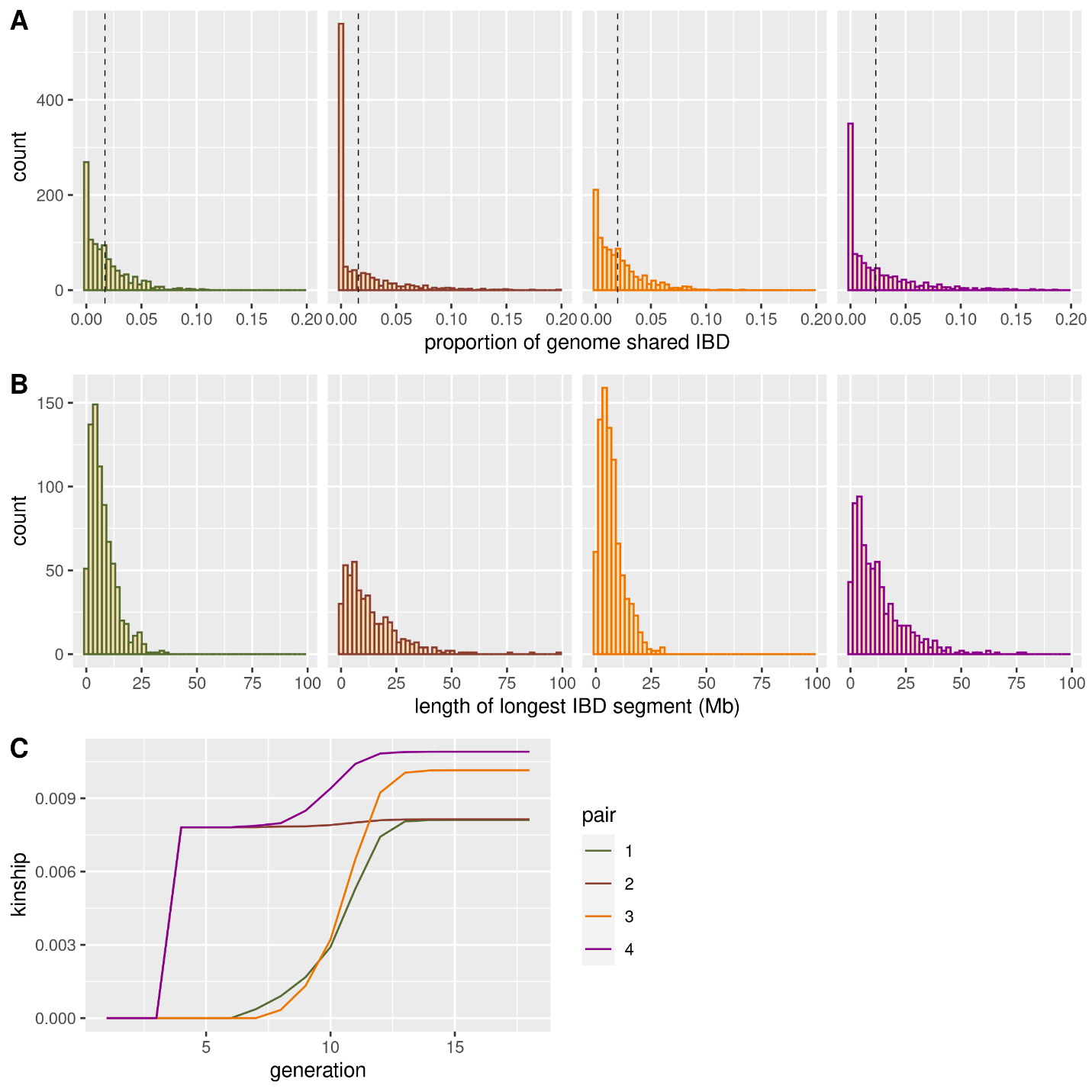


Supplementary Figure 6. IBD sharing for 4 arbitrarily selected pairs in the SLSJ genealogy. A: histogram of the proportion of the simulated segments shared IBD values over 1000 simulations (the mean is indicated by the dashed line). B: histogram of the length (in mega base pairs) of the longest IBD segment (for those simulations with non-zero sharing) C: Genealogical kinship by assumed founding generation for the 4 pairs of probands.


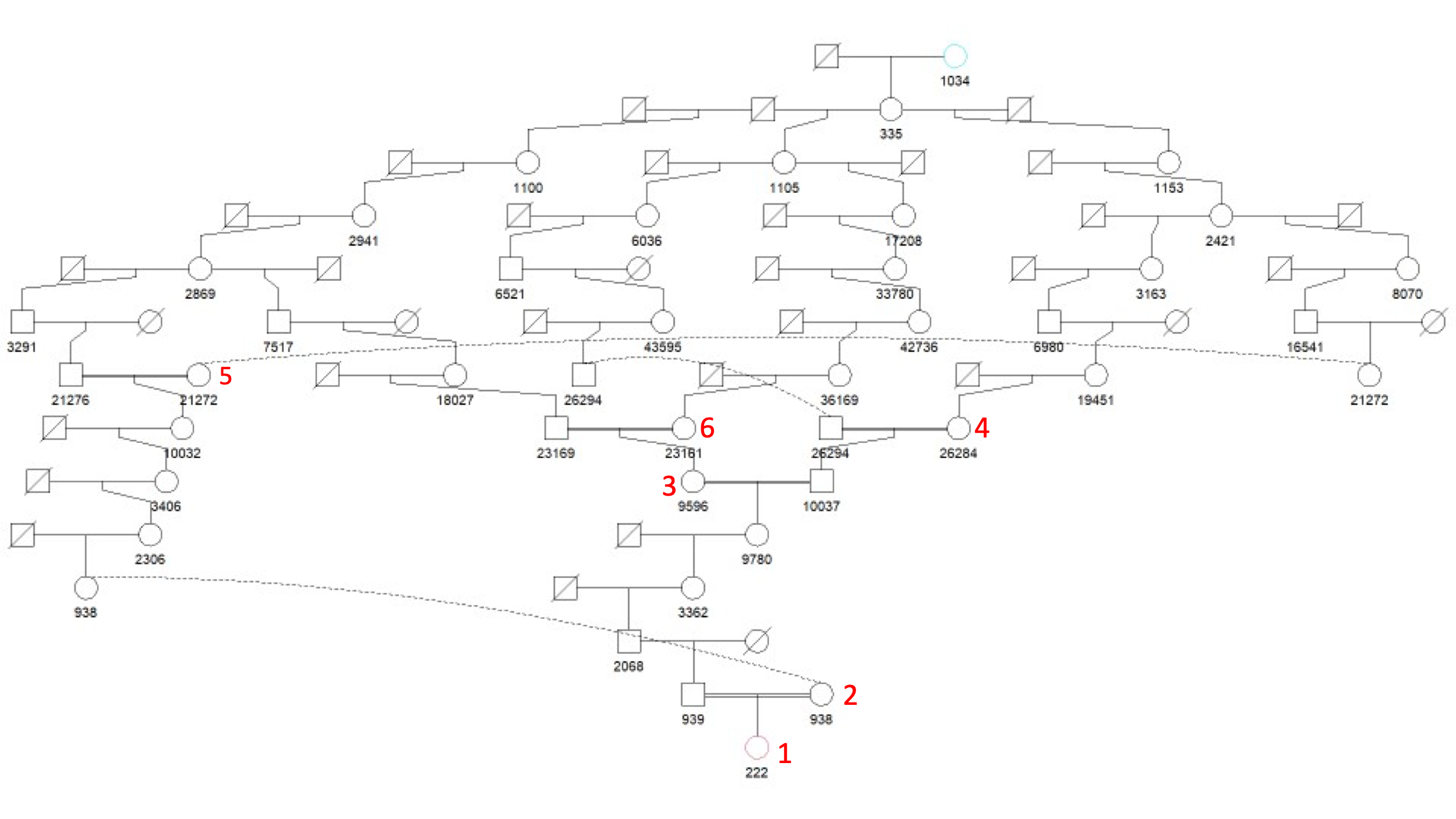


Supplementary Figure 7. Genealogy between proband “222” and ancestor “335”. There are 6 possible paths between “222” and “1034”, as labelled in the figure.

Of the 25,000 simulations, there were 4968 instances of a segment being inherited from ancestor “335”. Of those 4968 simulations, 4961 involved inheritance directly down a single path. The frequency of each path taken is shown in Supplementary Table 3. The remaining 7 occurrences were inherited through a ‘concatenation’ event, i.e.: multiple paths joined at an internal node. Paths 2 and 5 are shorter, leading to their increased prevalence. Even though the ‘concatenation’ event was quite rare, when it occurs it leads to a much longer segment (Supplementary Figure 8).

Supplementary Table 3. Frequency of each possible paths between proband “222” and ancestor “335” described in Supplementary Figure 7.

| Path1 | Path 2 | Path 3 | Path 4 | Path 5 | Path 6 |
| --- | --- | --- | --- | --- | --- |
| 650 | 1396 | 403 | 405 | 1401 | 436 |


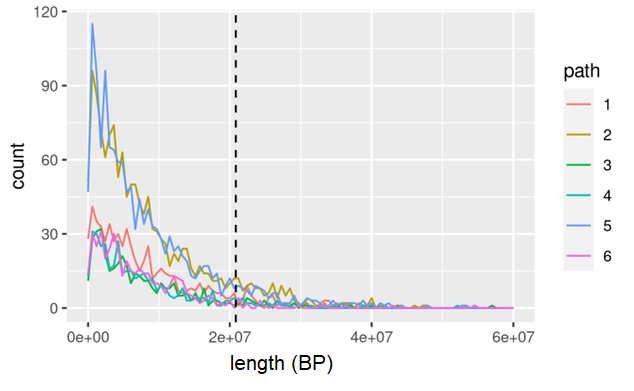


Supplementary Figure 8. Distribution of inherited segment lengths, depending on the path of inheritance between proband “222” and ancestor “335” described in Supplementary Figure 7. The vertical dashed line is the mean of the 7 segments that were inherited through concatenating segments at one of the internal ancestors.

**References**

Bherer, C., Campbell, C.L. and Auton, A. Refined genetic maps reveal sexual dimorphism in human meiotic recombination at multiple scales. *Nature Communications* 2017;8.

Boehnke, M. Limits of resolution of genetic linkage studies: Implications for the positional cloning of human disease genes. *American Journal of Human Genetics* 1994;55(2):379-390.

Broman, K.W. and Weber, J.L. Characterization of human crossover interference. *American Journal of Human Genetics* 2000;66(6):1911-1926.

Burkett, K.M.*, et al.* Correspondence Between Genomic- and Genealogical/Coalescent-Based Inference of Homozygosity by Descent in Large French-Canadian Genealogies. *Front Genet* 2022;12:808829.

Caballero, M.*, et al.* Crossover interference and sex-specific genetic maps shape identical by descent sharing in close relatives. *PLoS Genetics* 2019;15(12):1-29.

Eddelbuettel, D. and Francois, R. Rcpp: Seamless R and C plus plus Integration. *Journal of Statistical Software* 2011;40(8):1-18.

Haldane, J.B.S. The combination of linkage values, and the calculation of distances between the loci of linked factors. *Journal of Genetics* 1919;8(4):299-309.

Karlin, S. and Liberman, U. Classifications and comparisons of multilocus recombination distributions. *Proc Natl Acad Sci U S A* 1978;75(12):6332-6336.

Karlin, S. and Liberman, U. A natural class of multilocus recombination processes and related measures of crossover interference. *Advances in applied probability* 1979;11(3):479-501.

Laprise, C. The Saguenay-Lac-Saint-Jean asthma familial collection: the genetics of asthma in a young founder population. *Genes Immun* 2014;15(4):247-255.

Li, B., Wang, G.T. and Leal, S.M. Generation of sequence-based data for pedigree-segregating Mendelian or Complex traits. *Bioinformatics* 2015;31(22):3706-3708.

Nieuwoudt, C., Brooks-Wilson, A. and Graham, J. SimRVSequences: an R package to simulate genetic sequence data for pedigrees. *Bioinformatics* 2020;36(7):2295-2297.

Risch, N. and Lange, K. An alternative model of recombination and interference. *Ann Hum Genet* 1979;43(1):61-70.

Sturt, E. A mapping function for human chromosomes. *Annals of Human Genetics* 1976;40(2):147-163.

Varin, M.*, et al.* Age-Related Eye Disease and Participation in Cognitive Activities. *Sci Rep* 2017;7(1):17980.

Varin, M.*, et al.* Age-Related Eye Disease and Cognitive Function: The Search for Mediators. *Ophthalmology* 2020;127(5):660-666.

Vézina, H. and Bournival, J.S. An overview of the BALSAC database: past developments, current state and future prospects. *Historical Life Course Studies* 2020;11(2):1-17.
